# PCD Disseminates Public Health Interventions Addressing Chronic Disease Prevention and Health Promotion

**DOI:** 10.5888/pcd16.190123

**Published:** 2019-05-09

**Authors:** Leonard Jack

**Figure:**
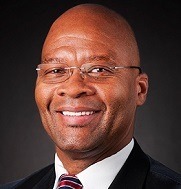
Leonard Jack Jr, PhD, MSc

We were fortunate to have a productive, innovative, and successful year in 2018 (1–8). In 2019 we want to build on that success and explore new ways to bring the best in public health research, policy, and practice to our readers. Last year saw the introduction of 2 new manuscript types — Implementation Evaluation and Program Evaluation Briefs — to help us better identify submissions that provide careful, thoughtful, and rigorously conducted research and evaluation findings derived from the application of population-based approaches.

This year PCD is continuing to review and evaluate the best article types and formats for sharing important work by identifying quality manuscripts that report findings from researchers and practitioners working in various settings. From the beginning, PCD’s mission has been to recognize the insights that can be gained outside of traditional research and evaluation models. We understand and appreciate that there are myriad ways practitioners, researchers, evaluators, and policy makers create and implement promising practices, from strategies and programs to trainings to system changes. With all of this in mind, PCD introduced a new article type in February: Public Health Practice Briefs. These articles highlight public health practices outside of traditional research and evaluation. We believe that the inclusion of a practice-focused article type fills a niche in the public health literature and gives potential PCD authors maximum flexibility in choosing the best format to showcase their work.

Last year PCD set a publication record, publishing 5 collections addressing a diversity of topics, from state and local public health actions to childhood obesity to health disparities and implementation evaluation, in addition to our regular collection devoted to PCD’s Student Research Paper Contest. Many of these topics were a response to PCD’s expert panel recommendations to expand and explore new public health topic areas. In 2019 PCD expects to break the record set in 2018, with more collections planned that continue to address the recommendations of PCD’s external review panel recommendations. Here are some of the highlights for 2019:

**CDC’s Colorectal Cancer Control Program.** Colorectal cancer is the second most common cause of cancer death among cancers that affect both men and women (1). The collection, expected in August, shares findings covering 2009 through 2015 from 25 states and 4 tribal organizations that were funded to implement evidence-based interventions to increase colorectal cancer screening rates in adults. This collection will include articles that describe the Colorectal Cancer Control Program, outcomes from the direct screening portion of the program, use of evidence based-interventions, and economic evaluation outcomes.

**Population health, place, and space: spatial perspectives in chronic disease research and practice.** Expected in October is PCD’s first collection sharing articles that highlight the use of geospatial information systems to increase understanding of the role of place and space in shaping the distribution of chronic disease to aid in identifying appropriate public health responses for chronic disease prevention and treatment. This PCD special collection documents research, translation, case studies, and analytic tools that have a spatial perspective and highlights the innovative and effective incorporation of place and space into chronic disease surveillance, prevention, and treatment.

**National Center for Chronic Disease Prevention and Health Promotion: programmatic efforts to reduce risk factors for chronic diseases.** The National Center for Chronic Disease Prevention and Health Promotion (NCCDPHP) works to reduce the risk factors for chronic diseases, especially for groups affected by health disparities, that is, differences in health across geographic, racial, ethnic, and socioeconomic groups (2). NCCDPHP has a rich portfolio of public health efforts to reduce the potential of developing chronic disease and to improve the health and quality of life for those living with chronic disease. PCD plans to publish later this year a collection on program evaluation efforts that offer insights into the development, implementation, and evaluation of population-based interventions to prevent chronic diseases and to control their effects on quality of life, morbidity, and mortality. Articles in this collection will be generated by NCCDPHP, its partners, and its awardees.

**Health care systems, public health, and communities: population health improvements.** Reducing the burden of chronic disease is a global challenge requiring diverse collaborations and diffusion and adoption of effective interventions in multiple settings. The past decade has seen a range of innovative community-driven and clinically driven primary and secondary prevention strategies designed to prevent and reduce the burden of chronic conditions worldwide. PCD plans to publish a collection on health care systems, public health, and communities that provides research, evaluation, and other work describing innovative and effective ways to link health care and community health to improve population health.

**Reducing obesity in high-obesity areas.** Obesity is a major public health challenge. In 2014, the Division of Nutrition, Physical Activity, and Obesity at the Centers for Disease Control and Prevention supported 11 land grant institutions in states that had at least one county with an adult obesity rate over 40%. The goal of this program was to implement evidence-based strategies at the state and community levels to improve environments for good nutrition and increased physical activity. This collection will contain articles providing information on partnerships, community participatory action, and programmatic activities implemented in counties with high rates of obesity among its adult populations.

**Good health and wellness in Indian country.** American Indians and Alaska Natives have a high rate of chronic diseases, which requires sustained culturally appropriate approaches to ameliorate. This collection will feature articles describing various aspects of the Good Health and Wellness in Indian Health Program — a culturally appropriate and coordinated program designed to implement chronic disease prevention and health promotion in American Indian and Alaska Native communities.

**Public Health and pharmacy: collaborative approaches to improve population health.** Reducing the burden of chronic disease is a global challenge requiring diverse collaborations and dissemination and adoption of effective interventions in multiple settings. Over the past decade a range of innovative community and clinical prevention strategies have been used in public health and pharmacy to prevent and reduce the burden of chronic conditions. Early this year, PCD announced a call for papers that provide timely information on effective ways the disciplines of public health and pharmacy can collaborate to improve the nation’s health and improve population health globally (https://www.cdc.gov/pcd/announcement.htm). PCD welcomes articles that describe collaboration among public health agencies, schools of pharmacy, community pharmacies, health care partners, and others to implement clinical and nonclinical strategies; approaches that pharmacies and pharmacists can use to improve population health; and ways health care and public health data can guide the integration of clinical and public health approaches to chronic conditions. Manuscripts for this collection are due to PCD on or before Friday, October 31, 2019.

We are enthusiastic about these collections and the opportunities they give us to delve deeper into these public health areas. We will keep our readers up to date on the progress of these collections and welcome any suggestions you may have for future collections. On behalf of all PCD staff members, editorial board members, associate editors, and peer reviewers, we thank you for interest in the work of the journal. We also thank the many authors who contributed to collections in 2018 and made the year such a successful one for the journal. We look forward to an exciting 2019 and to working closely with authors to continue our mission to publish relevant and timely articles of the highest quality.

## References

[1] Jack L Jr . Using PCD’s first-ever external review to enhance the journal’s worldwide usefulness to researchers, practitioners, and policy makers. Prev Chronic Dis 2018;15:E41. 10.5888/pcd15.180133 29625629 PMC5894299

[2] National Center for Chronic Disease Prevention and Health Promotion. https://www.cdc.gov/chronicdisease/index.htm. Accessed March 20, 2019.

[3] Jack L Jr . PCD advances recommendations from first-ever external review. Prev Chronic Dis 2018;15:E111. 10.5888/pcd15.180414 30191808 PMC6130284

[4] Jack L Jr . PCD increases content about public health approaches being implemented to improve population health. Prev Chronic Dis 2018;15:E164. 10.5888/pcd15.180644 30576270 PMC6307837

[5] State and local public health actions to prevent and control chronic diseases. 2018. https://www.cdc.gov/pcd/collections/pdf/PCD_StateAndLocal_Collection_FINAL_3-27-18.pdf. Accessed March 20, 2019.

[6] Student research paper contest. 2017. https://www.cdc.gov/pcd/collections/pdf/PCD_2017StudentPapersCombined.pdf. Accessed March 20, 2019.

[7] Carrie D , Brook B , Heidi B . The Childhood Obesity Research Demonstration (CORD) Project. https://www.cdc.gov/pcd/collections/pdf/CORD_Collection.pdf. Accessed March 20, 2019.

[8] Eliminating health disparities. 2018. https://www.cdc.gov/pcd/collections/Health_Disparities_Collection_2018.htm. Accessed March 20, 2019.

[9] Promoting the science and practice of implementation evaluation in public health. 2018. https://www.cdc.gov/pcd/collections/pdf/PCD_IE_Collection_final_Dec-2018.pdf. Accessed March 20, 2019. 10.5888/pcd15.180645 PMC6307834 30576271

